# Complexation and immobilization of arsenic in maize using green synthesized silicon nanoparticles (SiNPs)

**DOI:** 10.1038/s41598-024-56924-3

**Published:** 2024-03-14

**Authors:** Oyinade A. David, Ayomide H. Labulo, Ibrahim Hassan, Idowu Olawuni, Charles O. Oseghale, Augustine D. Terna, Olamilekan O. Ajayi, Samuel A. Ayegbusi, Michael O. Owolabi

**Affiliations:** 1 Department of Plant Science and Biotechnology, Federal University Oye-Ekiti, Oye-Ekiti, Ekiti State Nigeria; 2 Department of Chemistry, Federal University of Lafia, Lafia, Nasarawa State Nigeria; 3 Department of Biochemistry, Obafemi Awolowo University, Ile-Ife, Osun-State Nigeria; 4 Department of Chemistry, Federal University of Technology, Owerri, Imo State Nigeria; 5 Plant Environmental Signalling and Development, Faculty of Biology, University of Freiburg, 79104 Freiburg, Germany; 6 CIBSS (Centre for Integrative Biological Signalling Studies), University of Freiburg, 79104 Freiburg, Germany

**Keywords:** Arsenic toxicity, Immobilisation, Maize growth and yield, Phytostabilization, Silicon nanoparticles, Biochemistry, Plant sciences

## Abstract

Arsenic (As) is a heavy metal that is toxic to both plants and animals. Silicon nanoparticles (SiNPs) can alleviate the detrimental effects of heavy metals on plants, but the underlying mechanisms remain unclear. The study aims to synthesize SiNPs and reveal how they promote plant health in Arsenic-polluted soil. 0 and 100% v/v SiNPs were applied to soil, and Arsenic 0 and 3.2 g/ml were applied twice. Maize growth was monitored until maturity. Small, irregular, spherical, smooth, and non-agglomerated SiNPs with a peak absorbance of 400 nm were synthesized from *Pycreus polystachyos*. The SiNPs (100%) assisted in the development of a deep, prolific root structure that aided hydraulic conductance and gave mechanical support to the maize plant under As stress. Thus, there was a 40–50% increase in growth, tripled yield weights, and accelerated flowering, fruiting, and senescence. SiNPs caused immobilization (As(III)=SiNPs) of As in the soil and induced root exudates Phytochelatins (PCs) (desGly-PC_2_ and Oxidized Glutathione) which may lead to formation of SiNPs=As(III)–PCs complexes and sequestration of As in the plant biomass. Moreover, SiNPs may alleviate Arsenic stress by serving as co-enzymes that activate the antioxidant-defensive mechanisms of the shoot and root. Thus, above 70%, most reactive ROS (OH) were scavenged, which was evident in the reduced MDA content that strengthened the plasma membrane to support selective ion absorption of SiNPs in place of Arsenic. We conclude that SiNPs can alleviate As stress through sequestration with PCs, improve root hydraulic conductance, antioxidant activity, and membrane stability in maize plants, and could be a potential tool to promote heavy metal stress resilience in the field.

## Introduction

Arsenic (As) is a toxic metalloid that poses a serious threat to human health and agricultural productivity. Contamination of soil and water resources is a global problem, affecting millions of people and several crop species^1^. As can enter the plant system through the roots and interfere with various physiological and biochemical processes, such as photosynthesis, respiration, nutrient uptake, water balance, and antioxidant defense^2^. As a result, stress can cause reduced plant growth, chlorosis, necrosis, oxidative damage, and ultimately plant death^3^. Moreover, As can accumulate and translocate in plant tissues, especially in the edible parts, posing a risk to food safety and human health^4^.

Maize (*Zea mays* L.) is one of the most important cereal crops in the world, providing food, feed, and biofuel for millions of people. However, maize is sensitive to As stress and shows adverse effects on its growth, development, yield, and quality when exposed to high levels of As in the soil or irrigation water^5^. Therefore, it is essential to find effective strategies to mitigate As toxicity and accumulation in maize plants and enhance their tolerance and adaptation to As stress.

One of the strategies to cope with As stress is the synthesis of phytochelatins (PCs), which are low molecular weight cysteine-rich peptides that can bind and sequester As in plant cells^5^. PCs are synthesized by the enzyme phytochelatin synthase (PCS) from glutathione (GSH) or its precursor γ-glutamylcysteine (γ-EC) in response to As exposure^6^. PCs can form complexes with As (III) or As (V) and transport them to the vacuoles for detoxification. PCs can also modulate the redox status of the cells by maintaining the GSH/GSSG ratio and enhancing the antioxidant defense system^7^. The synthesis and characterization of PCs in plants under As stress have been studied by various methods, such as high-performance liquid chromatography (HPLC), mass spectrometry (MS), nuclear magnetic resonance (NMR), and fluorescence microscopy^8^

Another strategy to cope with As stress is the application of silicon (Si), which is a beneficial element for plants that can improve their growth, development, and resistance to various biotic and abiotic stresses, such as drought, salinity, heavy metals, and pathogens^9^. Si can be applied to plants in different forms, such as soluble silicates, silicic acid, or silicon nanoparticles (SiNPs). SiNPs are nanosized particles of silicon dioxide (SiO_2_) that have unique physical and chemical properties, such as high surface area, high reactivity, high stability, and easy penetration into plant cells^10^. SiNPs have been shown to enhance plant growth, photosynthesis, nutrient uptake, water use efficiency, and stress tolerance in various crops, such as rice, wheat, tomato, and lettuce^10^.

Several studies have reported the beneficial effects of SiNPs on alleviating As toxicity in plants by reducing As uptake and translocation, increasing As sequestration and detoxification, modulating physiological and biochemical responses, and enhancing antioxidant defense mechanisms^11^. However, the role of SiNPs in mitigating As stress in maize plants has not been well explored. Moreover, the interaction between SiNPs and PCs in maize plants under As stress has not been investigated. Therefore, the aim of this study was to characterize the PCs synthesized by maize plants under As stress and to evaluate the physiological effects of SiNPs on maize plants grown in As-contaminated soil.

The specific objectives of this study were to synthesize SiNPs and evaluate the effects of SiNPs on maize growth, yield, and physiology under As stress and to quantify and characterize PCs in maize roots under As stress using HPLC–MS.

## Materials and methods

### Synthesis and characterization of SiNPs

#### Plant and collection guidelines

A silicon-rich precursor, *Pycreus polystachios*, commonly referred to as bunchy flat sedge, was used for the synthesis of silicon nanoparticles. *Pycreus polystachyos* shoots were collected at Federal University Lafia (FULafia), Nigeria (8.4787° N, 8.5572° E). The plants were identified at the Herbarium, Plant Science and Biotechnology, FULafia (Herbarium voucher number: 049), Nigeria. According to the IUCN (2018), *Pycreus polystachyos* belongs to the family Cyperaceae and is native to sub-Saharan Africa, among other countries. Assessment information revealed that *Pycreus polystachyos* belongs to the red list category of least concern as it’s widespread with a stable population without being threatened. The experimental research and field studies on plants (either cultivated or wild), including the collection of plant material, were done with relevant institutional, national, and international guidelines and legislation.

#### Extraction of SiNPs

The plant leaves (500 g) were collected, rinsed well with distilled water, and heated on a heating mantle for an hour. A Whatman filter was used to filter the solution. The filtrate was then exposed to pyrolysis at a high temperature (200 °C) in a common microwave oven for 40 min, resulting in a pale-yellow solution^12^. The sample was stored in a refrigerator at 4 °C until further use. The SiNPs were characterized using a UV spectrophotometer, scanning electron microscope (SEM), EDX, and FTIR spectra.

### Soil collection and planting preparation

#### Soil collection

The soil collected was sandy loam, pH 5.6, organic carbon 4.07%, available nitrogen 0.4%, and available phosphorus 16 mg/Kg. Sterilized seeds were planted into plastic pots containing 6 kg of soil. Pots were arranged in a completely randomized design with three replicates in the screen house, Department of Plant Science and Biotechnology (7°80″ N, 5°21″ E), Federal University Oye-Ekiti, Ekiti State, Nigeria, with temperatures ranging from 21 to 28 °C. 2.2.2 Planting preparation Maize Variety Pro-vitamin Seeds were obtained from the Institute of Agricultural Research and Technology (IAR&T), Apata, Ibadan, Nigeria. The seeds were surface sterilized with 15% sodium hypochlorite, rinsed with distilled water, and air-dried before planting.

#### Application of SiNPs

Two weeks after planting, 25 mL of 100% v/v SiNPs (S100) were applied once to the soil and other soils received water (S0) of the same volume. Afterwards, 150 mL of 0 (As0) and 3.2 g/mL (As3.2) of Arsenic trioxide were applied after 3 weeks of planting. The Arsenic trioxide application was repeated after another 3 weeks. A concentration of 25 mL of 100% v/v green synthesized SiNPs was adopted because it is non-toxic and biodegradable^13^ and it has been reported to be the optimal concentration of SiNPs solution for phytoremediation of most toxic metals (e.g. Cd, As, etc.) in contaminated soil^14^. Higher volumes of SiNPs solution (50 mL or 75 mL) had negative effects on plant growth and metal tolerance^14^. These concentrations were chosen based on previous studies that showed the beneficial effects of SiNPs on plant growth and stress tolerance^15^. Arsenic trioxide concentrations were selected based on preliminary tests that determined the optimal and suboptimal dosage effects on plants. Previous studies that used similar concentrations of Arsenic trioxide in plants were also consulted^16^. SiNPs were applied two weeks after planting, and Arsenic trioxide was applied three weeks after planting. The Arsenic trioxide application was repeated after another three weeks. Soils were watered to the field's moisture capacity. The plant was monitored till maturity (60 days).

### Biochemical analysis

The morphological features of maize plants, such as plant height, stem diameter, leaf length, number of falling leaves, root and shoot weight, root/shoot ratio, weight of fruit (husks and grains), and floral characteristics (flag and spike length and flowering days), were measured using standard methods. A total sample population (N) of 36 maize plants was harvested at the maturity stage (75 days after sowing). The plants were separated into roots, shoots, leaves, fruit (husks and grains. The roots were washed thoroughly with tap water and then with distilled water to remove soil particles.

The antioxidant enzyme activities of maize plants, such as superoxide dismutase (SOD), and catalase (CAT), in the shoots were determined by spectrophotometry following the method described by Verma et al*.*^10^. About 0.5 g of fresh plant tissue was homogenized with 50 mM phosphate buffer (pH 7.0) containing 1 mM EDTA and 1% polyvinylpyrrolidone, and the homogenate was centrifuged at 15,000 rpm for 15 min. The supernatant was used as an enzyme extract^17^. The CAT activity was measured by monitoring the decomposition of hydrogen peroxide at 240 nm using a spectrophotometer. A spectrophotometer was used to measure the SOD activity by keeping an eye on how well it stopped the reduction of nitroblue tetrazolium (NBT) at 560 nm^18^. The redox status of maize plants, such as reduced glutathione (GSH), oxidized glutathione (GSSG), and GSH/GSSG ratio in the shoots were determined by an enzymatic recycling method following the method described by^19^. About 0.5 g of dried plant powder was extracted with 5% sulfosalicylic acid, and the extract was centrifuged at 10,000 rpm for 15 min. The supernatant was used for GSH and GSSG estimation. For the GSH estimation, 100 μL of the supernatant was mixed with 900 μL of reaction buffer (0.1 M phosphate buffer, pH 7.5, containing 5 mM EDTA), and the absorbance was measured at 412 nm using a spectrophotometer as the blank value^20^.

For the GSSG estimation, 100 μL of the supernatant was mixed with 900 μL of reaction buffer containing NADPH (nicotinamide adenine dinucleotide phosphate, reduced form, 0.3 mM) and GR (30 U mL^−1^) and incubated at room temperature for 30 min. Then, 10 μL of DTNB (10 mM) was added to the mixture, and the absorbance was measured at 412 nm after 5 min. The GSSG content was calculated using the following formula:

2 $$GSSG \left(\mu {\rm mol}{{\rm g}}^{-1}\right)= \frac{[\left({A}_{s}- {A}_{b}\right) \times {V}_{t}]}{(\varepsilon \times {V}_{r } \times W \times 2)}$$

where A_s_ is the sample absorbance, A_b_ is the blank absorbance, V_t_ is the total volume, ε is the molar extinction coefficient of TNB (13.6 mM^−1^ cm^−1^), V_r_ is the volume of extract used, W is the weight of plant tissue, and 2 is the factor for two GSH molecules per GSSG molecule.

The GSH/GSSG ratio was calculated by dividing the GSH content by the GSSG content.

The synthesis of phytochelatins (PCs) in maize roots was determined by high-performance liquid chromatography (HPLC) following the method described by^21^. About 10 g of dried root powder was extracted with 20 mL of acetonitrile in methanol, and the extract was centrifuged at 10,000 rpm for 15 min. The supernatant was filtered through a 0.22 μm membrane filter, and 10 µL of sample was injected into an HPLC (Shimadzu (Nexera MX)) system equipped with a C18 column and a UV detector. The mobile phase consisted of 0.1 M sodium acetate buffer (pH 4.5) containing 5 mM EDTA and methanol (95:5 v/v) at a flow rate of 1 mL min^−1^. The PCs were detected at 254 nm and quantified by comparing them with standard curves prepared with known concentrations of glutathione (GSH), phytochelatin-2 (PC_2_), phytochelatin-3 (PC_3_), and phytochelatin-4 (PC_4_).

The phenols and flavonoids contents in maize roots were determined by colorimetric methods following the method described by^22^ About 0.5 g of dried root powder was extracted with 80% methanol, and the extract was centrifuged at 10,000 rpm for 15 min. The supernatant was used for phenol and flavonoid estimation. For the phenol estimation, 500 μL of the supernatant was mixed with 2.5 mL of Folin–Ciocalteu reagent (diluted ten times with distilled water) and incubated at room temperature for 5 min. Then, 2 mL of sodium carbonate solution (7.5%, w/v) was added to the mixture and incubated at room temperature for 90 min. The absorbance of the mixture was measured at 765 nm using a spectrophotometer. The phenol content was calculated using a standard curve prepared with known concentrations of gallic acid.

For the flavonoids estimation, 500 μL of the supernatant was mixed with 1.5 mL of distilled water, and then 75 μL of sodium nitrite solution (5%, w/v) was added to the mixture and incubated at room temperature for 6 min. Then, 150 μL of aluminum chloride solution (10% w/v) was added to the mixture and incubated at room temperature for another 6 min. Then, 500 μL of sodium hydroxide solution (1 M) was added to the mixture and diluted with distilled water to make up 2.5 mL. The absorbance of the mixture was measured at 510 nm using a spectrophotometer. The flavonoid content was calculated using a standard curve prepared with known concentrations of quercetin.

We found out how well maize shoots get rid of hydroxyl radicals by using the deoxyribose degradation method, which is similar to the method Tripathi et al.^23^. About 0.5 g of dried root powder was extracted with 80% methanol, and the extract was centrifuged at 10,000 rpm for 15 min. The reaction mixture was incubated at 37 °C for 1 h, and then 1 mL of thiobarbituric acid (TBA) solution (1%, w/v) and 1 mL of trichloroacetic acid (TCA) solution (2.8%, w/v) were added to the mixture and heated in a boiling water bath for 15 min. The absorbance of the mixture was measured at 532 nm using a spectrophotometer. The hydroxyl radical-scavenging activity was calculated using the following formula:

3 $$Hydroxyl\,radical\,scavenging\,activity \left(\%\right)= \frac{({A}_{c}- {A}_{s})}{{A}_{c}} \times 100$$

where A_c_ is the absorbance of the control without the extract and A_s_ is the absorbance of the sample with the extract.

The lipid peroxidation and IC_50_ of maize roots were determined by the thiobarbituric acid reactive substances (TBARS) method following the method described by^24^ About 0.5 g of dried root powder was extracted with 10 mL of trichloroacetic acid (TCA) solution (0.1%, w/v) and the extract was centrifuged at 10,000 rpm for 15 min. The supernatant was used for malondialdehyde (MDA) and IC_50_ estimation. For MDA estimation, 2 mL of the supernatant was mixed with 2 mL of TBA solution (0.5%, w/v) and heated in a boiling water bath for 30 min. The absorbance of the mixture was measured at 532 nm and corrected for non-specific absorption at 600 nm using a spectrophotometer. The MDA content was calculated using the following formula:

4 $$MDA \left({\rm nmol}{{\rm g}}^{-1}\right)= \frac{[\left({A}_{532}- {A}_{600}\right) \times {V}_{t}]}{(\varepsilon \times {V}_{r} \times W)}$$

To find the IC_50_, different amounts of sodium arsenate (0, 0.5, 1, 2, 4, and 8 mM) were added to the supernatant and left to sit at room temperature for 30 min. Sodium arsenate was used instead of Arsenic trioxide because sodium arsenate has a higher IC_50_ value than Arsenic trioxide, meaning that it is less toxic to the cells at the same concentration. The MDA content was then measured as described above. The IC_50_ was calculated as the concentration of sodium arsenate that caused a 50% inhibition of MDA production.

### Determination of arsenic in soil and plant

The samples were dried properly in the oven at 50 °C. The dried samples were ground into a fine powder, and each sample was weighed into a Teflon beaker. 20 mL of freshly prepared aqua-regia (HCl: HNO_3_ (3:1)) was added to the grinded samples and heated to near dryness. After heating, 20 mL of distilled water was added. Filtered after second heating, the filtrate was made up to mark in a 25 mL flask and kept refrigerated pending total metal determination by AAS.

### Statistical analysis

The data were subjected to analysis of variance (ANOVA) using SPSS software (version 16.0). The mean values were compared by Duncan’s multiple range test (DMRT) at *p* < 0.05.

## Results and discussion

### Characterization of silicon nanoparticles (SiNPs) from *Pycreus polystachyos*

**Figure 1:**
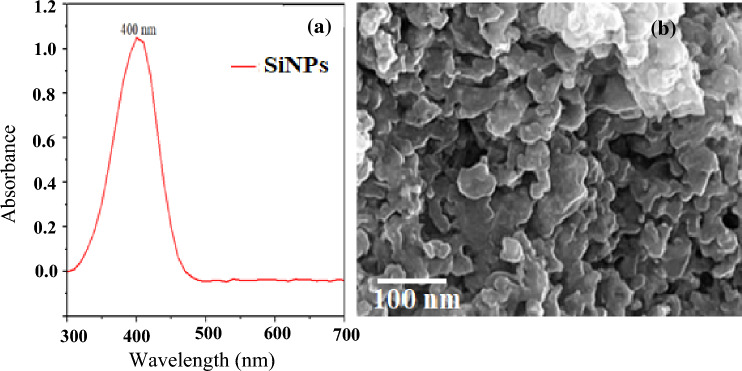
(**a**) UV–Vis spectra of the synthesized SiNPs, (**b**) SEM image of synthesized SiNPs using *Pycreus polystachyos.*

Nano-silicon has become an effective tool in promoting plant development, resistance, and survival under abiotic stress^25^. The study aimed at the synthesis of SiNPs from *Pycreus polystachyos* and, consequently, its effect on the physiological and biochemical activities of maize plants under As stress. Figure 1a shows a graph of absorbance against wavelength for SiNPs. The peak at around 400 nm indicates the presence of SiNPs in the sample. SiNPs have a unique surface plasmon resonance (SPR) band in the visible range. This band is caused by the collective oscillation of free electrons on the nanoparticles' surface. The SPR band depends on the size, shape, and distribution of the nanoparticles, as well as the surrounding medium. High peak absorbance means a higher concentration of SiNPs, while average peak width means a more uniform size distribution of SiNPs.

**Figure 2:**
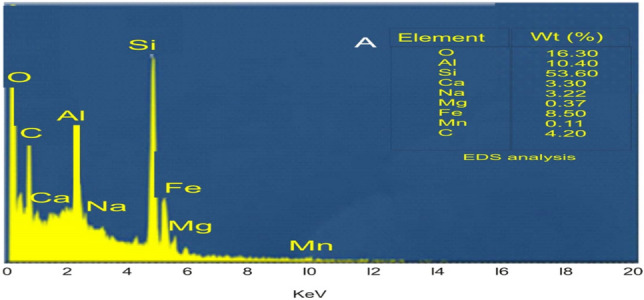
EDX spectra of synthesised SiNPs using *Pycreus polystachyos.*

Figure 1b shows the surface made up of small, irregularly shaped particles. These are the silica nanoparticles, which are spherical, non-agglomerated, and have a smooth surface morphology^26^ and the EDX spectra showed 53% silicon (Fig. 2). The average particle size of powdered SiNPs is about 40 nm (nm)^26^. The particles are densely packed and have a rough texture. This could indicate that the sample was prepared by compressing or sintering the powdered SiNPs. The rough texture could also be due to the noise or artifacts in the image.

**Figure 3:**
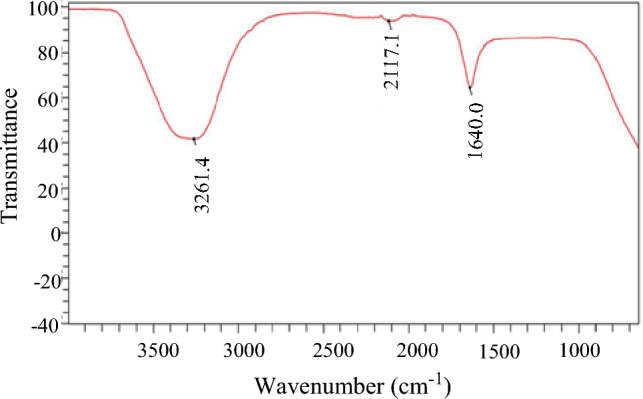
FTIR spectra of green synthesized SiNPs.

The FTIR spectra of the green synthesized SiNPs as depicted in Fig. 3 show three peaks at approximately 3260, 2170, and 1600 cm^−1^ respectively. These peaks correspond to the stretching vibrations of different functional groups on the surface of SiNPs. The peak at 3260 cm^−1^ is assigned to the O–H stretching vibration of hydroxyl groups (–OH) or water molecules adsorbed on SiNPs. The peak at 2170 cm^−1^ is assigned to the Si–H stretching vibration of silane groups (–SiH) or hydrogen atoms bonded to SiNPs. The peak at 1600 cm^−1^ is assigned to the C=O stretching vibration of carboxyl groups (–COOH) or organic molecules attached to SiNPs^27^,^28^. The spectrum shows two valleys at approximately 2750 and 1900 cm^−1^. These valleys correspond to the absorption bands of different functional groups on the surface of SiNPs. The valley, at 2750 cm^−1^ is assigned to the C–H stretching vibration of alkyl groups (–CH) or organic molecules attached to SiNPs. The valley at 1900 cm^−1^ is assigned to the C=C stretching vibration of alkenyl groups (–C=C) or organic molecules attached to SiNPs.

### The effects of SiNPs on maize growth under As stress

**Figure 4:**
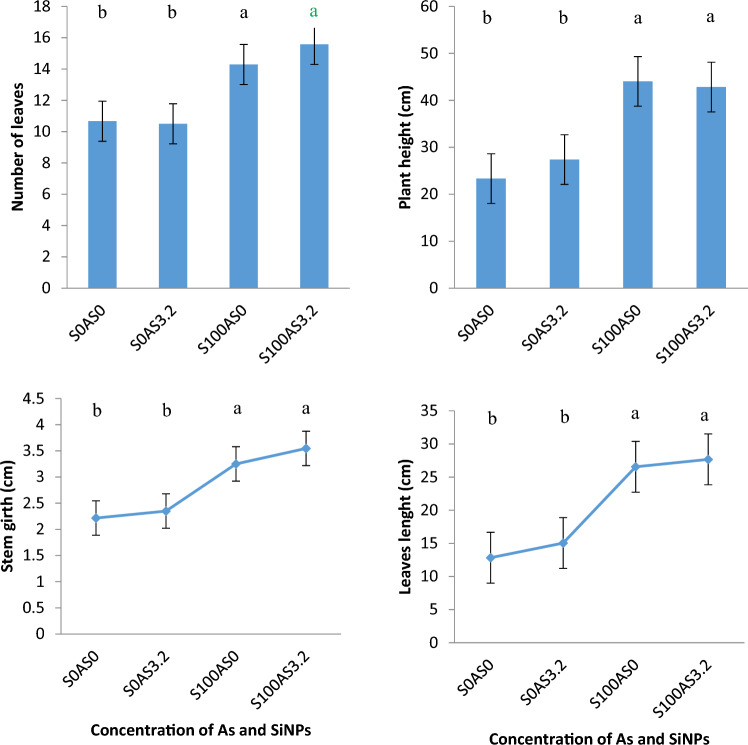
Morphological Features of Maize in Arsenic Soil subjected to SiNPs at 15 WAP. Bars represent error bars at *p* ≤ 0.05, data that do not share a letter are significantly different (*p* < .05, DMRT), N = 36.

The effects of SiNPs on maize growth under As stress are shown in Fig. 4. It can be seen that As stress had no significant reduction in the number of leaves, stem diameter, or leaf length of maize plants compared to the control (S0AS0). However, plant height was significantly sensitive to the treatment of As. These results indicate the role of C4 photosynthetic pathways in reducing photo-oxidation respiration in plants under stress. Application of SiNPs improved the development (40–55% increase) of maize plants under As stress compared to the As-stressed plants without SiNPs (S0AS3.2). These significant changes observed in the growth and development of maize under the SiNPs treatment could be attributed to the enhancement of cell wall strength, water uptake, nutrient absorption, and photosynthetic efficiency by SiNPs in maize plants under As stress^29^. Silicon was also known to improve the hypocotyl length, stem diameter, and flowering rate of *Vicia faba*^30^. This higher growth was more pronounced at S100As3.2 than in maize plants without As (S100As0). This could be due to the chemical reactivity between SiNPs and Arsenic, which assists in reducing its bioavailability. The significant effects of Si on maize growth can be associated with a protective layer formed by Si deposition, precipitation with heavy metal ions, and metabolism alteration under stress^31^.

### The floral characteristics of maize in arsenic polluted soil-using SiNPs

**Figure 5:**
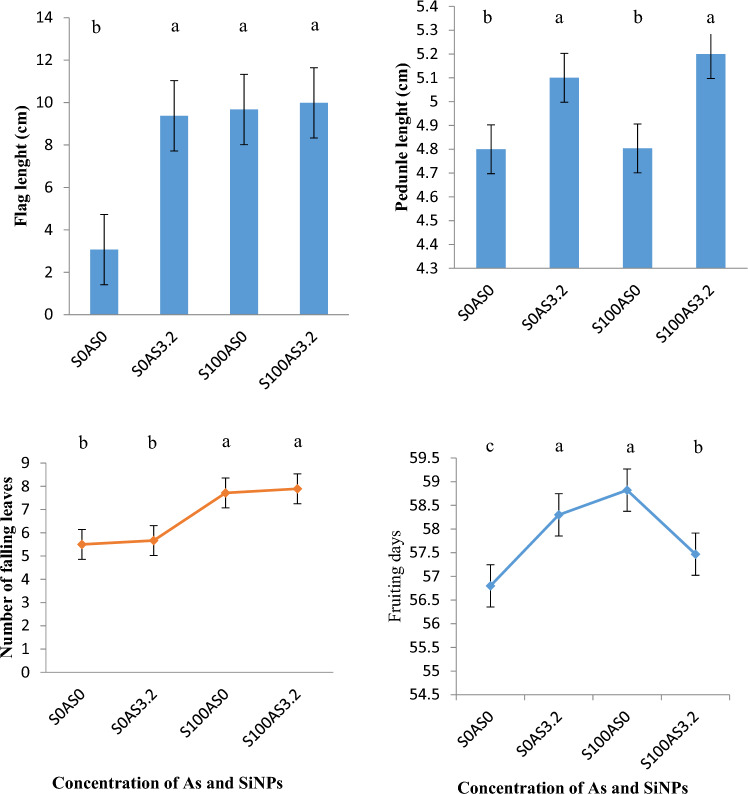
Floral Characteristics of Maize Planted in Arsenic Polluted Soil under SiNPs at 15 WAP. Bars represent error bars at *p* ≤ 0.05, data that do not share a letter are significantly different (*p* < .05, DMRT), N = 36.

Figure 5 shows the floral characteristics of maize plants grown in As-contaminated soil with or without SiNPs application. As stress (S0As3.2) initiated flowering traits (spike and flag length) and quickened days of fruiting compared to control (S0As0). These results indicate that As stress affected the reproductive development and yield potential of maize plants by affecting their flower initiation, pollination, fertilization, and fruit formation^32^. Application of SiNPs (S100As3.2) hastened flowering buds and fruiting periods under As stress. Similarly, SiNPs showed an increased number of flowers, flower diameter, and dry weight of flowers^33^. The improvement in floral characteristics under the SiNP treatment could be attributed to the enhancement of reproductive hormones and pollen viability in maize plants under As stress^32^. Without stress (S100As0), SiNPs delayed fruiting in maize. The number of falling leaves after 15 WAP in maize plants treated with SiNPs (S100) is significantly higher than in plants without (S0) SiNPs. This indicates that SiNPs induce aging (senescence) in maize plants with or without As stress.

### The yield and root development of Maize in Arsenic contaminated soil-using SiNPs

**Figure 6:**
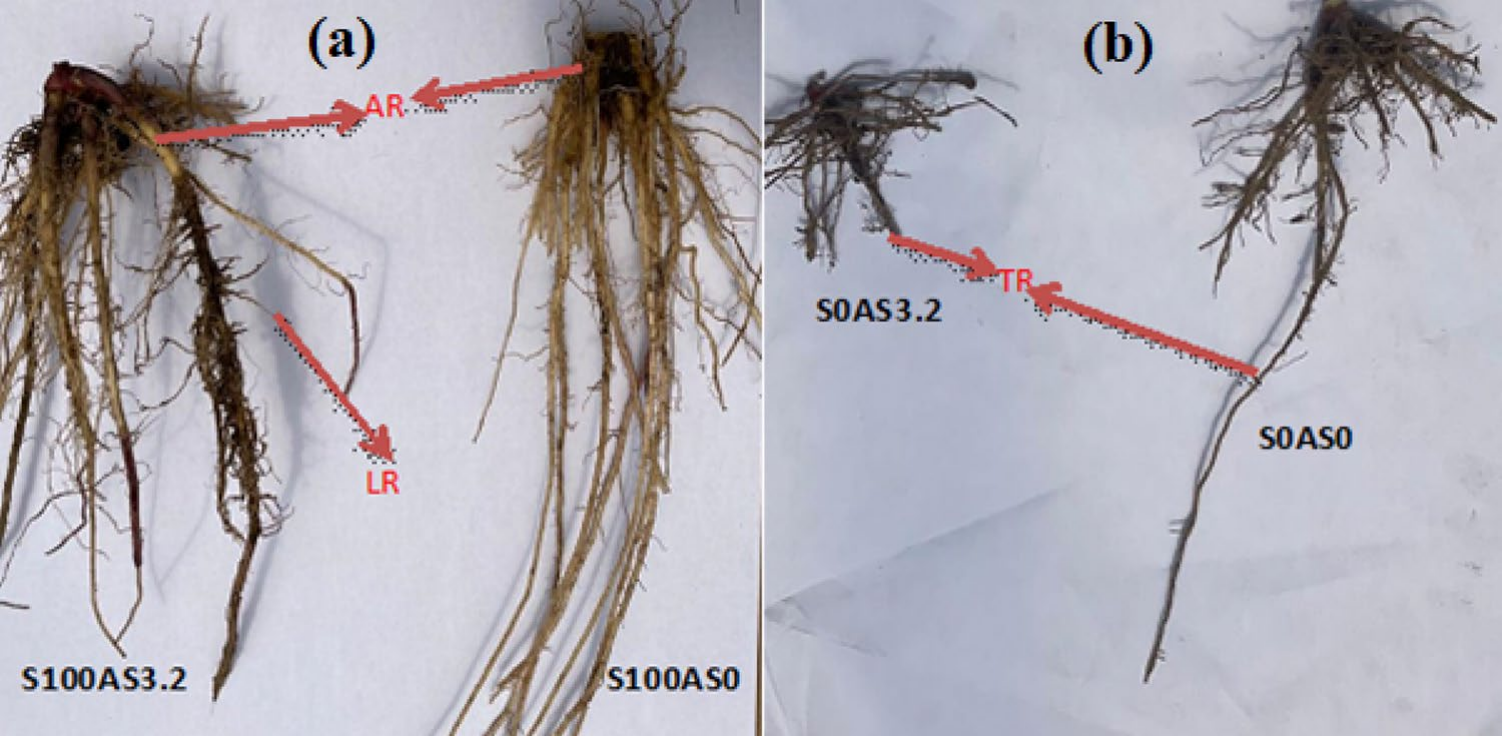
Architectural structure of maize root grown in Arsenic-polluted soil under SiNPs at 15 WAP. (**a**) LR = Lateral root, AR = Adventitious root and (**b**) TR = Taproot.

Table 1 and Fig. 6 show the yield and root structure of maize plants grown in As-contaminated soil with or without SiNPs application. There was no significant difference in the weight of the fruit, root, and shoot of the S0As3.2 maize plant compared to the control (S0AS0). However, the application of SiNPs tripled the weight of fruit, weight of root, weight of shoot, and root/shoot ratio with or without As stress compared with plants without SiNPs. SiNPs increase carbon assimilation, nutrient translocation, and energy allocation in maize. SiNPs assisted in significant root development; Fig. 6 showed a well-developed deep tap root with profusely branched adventitious roots, which facilitated the easy movement of nutrients in maize treated with SiNPs (S100) with or without As stress. On the other hand, the root of the plant exposed to As was short and shallow. The presence of prolific deep root structures enhanced water absorption compared to shallow-rooted S0As3.2. Changes in lateral root formation improve the plasticity of maize and other crops to environmental stress^34^. The functionality and structure of the root system can improve the effectiveness of plant hydraulics in releasing water to the leaves^35^. Si plays an important role in the regulation of the transpiration rate and hydraulic conductance of roots^36^. The root shoot ratio was high in plants treated with SiNPs (S100As0) compared to control (S0As0). Silicon helps to increase the root-to-shoot ratio^37^. Silicon nanoparticles help plants invest more in roots, and the obvious image (Fig. 6) shows the stronger root architecture of maize than plants without silicon.

**Table 1:** Yield of Maize grown in Arsenic-polluted soil under Silicon Nanoparticles at 15 WAP.

Treatment	Weight of Fruit (g)	Weight of root (g)	Weight of shoot (g)	Root/shoot ratio
S0AS0	5.205b	3.103b	28.54b	0.11027ab
S0AS3.2	5.693b	2.958b	33.08b	0.0746b
S100As0	16.890a	10.480a	87.41a	0.12852a
S100As	17.597a	8.404a	85.98a	0.09881ab

Mean values with the same letter along the same column are not significantly different at *p* ≤ 0.05.

### Effects of silicon nanoparticles on photosynthetic pigmentation and root secondary metabolites under As stress

The role of photosynthetic pigments such as chlorophyll contents and carotenoids is vital in carbon fixation, as they are involved in capturing solar radiation to drive the photosynthetic mechanism^38^. Arsenic stress reduced chlorophyll a, b, and total carotene compared to control (Table 2). Silicon nanoparticles increased 14–20% chlorophyll B in plants with or without As stress. Compared to other treatments, silicon nanoparticle (SiNPs) treatment under Arsenic (As) stress (S100As3.2) did not have a big effect on the levels of chlorophyll A and total carotene. Accumulation of anthocyanin during water stress tendered a multitude of roles, which include radical scavengers, photo-defendants, and signaling factors^39^. Table 3 shows the phenols and flavonoid contents of maize roots grown in As-contaminated soil with or without SiNPs application. It can be seen that As stress significantly reduced the phenols and flavonoid contents in the root tissues of maize plants, though not significantly compared to the control (S0AS0). These results indicate that As stress inhibited the synthesis of phenols and flavonoids in root cells^40^. The drop in phenols and flavonoids levels under As stress could be because ROS or direct interaction with As destroy precursors or enzymes that are needed to make them^40^.The application of SiNPs (S100As0) had no significant effect on the phenolic and flavonoid contents in the root tissues of maize plants. However, the significant decrease observed in phenolics and flavonoids under S100As3.2 could be a result of the toxicity of As to secondary metabolites.

**Table 2:** Effect of silicon nanoparticles on the photosynthetic pigment of maize root grown in Arsenic polluted soil.

Treatments	CHL A	CHL B	Total carotene
S0AS0	3.06 ± 0.032a	5.95 ± 0.026b	299.43 ± 2.55a
S0AS3.2	2.20 ± 0.038a	4.88 ± 0.586c	227.26 ± 2.24b
S100As0	2.78 ± 0.042a	7.12 ± 0.045a	226.12 ± 2.75b
S100As3.2	2.65 ± 0.029a	5.57 ± 0.041b	204.49 ± 1.72b

The mean values with the same letter along the same column are not significantly different at *p* ≤ 0.05.

**Table 3:** Effect of silicon nanoparticles on maize’s root secondary metabolite grown in arsenic polluted soil.

Treatment	Phenols (mg gae/g sample)	Flavonoids (mg que/g sample)
S0AS0	0.708 ± 0.04a	0.412 ± 0.006a
S0AS3.2	0.624 ± 0.007a	0.282 ± 0.009a
S100As0	0.652 ± 0.068a	0.299 ± 0.001a
S100As3.2	0.503 ± 0.029b	0.172 ± 0.025b

The mean values with the same letter along the same column are not significantly different at *p* ≤ 0.05.

### Effects of SiNPs on antioxidants in maize plants under As stress

Table 4 shows the antioxidant enzyme activities of maize plants grown in As-contaminated soil with or without SiNPs application. It can be seen that As stress significantly reduced the activities of SOD and CAT in the shoots of maize plants compared to the control (S0AS0). These results indicate that As stress-induced oxidative stress generated reactive oxygen species (ROS) that exceeded the antioxidant capacity of maize plants^41^. The decrease in antioxidant enzyme activities under As stress could be because ROS or direct interactions with As stop or break down these enzymes^42^. Upon the application of SiNPs, the activities of SOD and CAT dramatically showed an almost 100% increment in the shoots of maize plants under As stress (S100As3.2) compared to the As-stressed plants without SiNPs (S0AS3.2). SiNPs also behave like co-enzymes by activating antioxidant enzymes like CAT and SOD, which scavenge the reactive oxygen species ROS^42^.

**Table 4:** Antioxidant enzymes of Maize grown in Arsenic-polluted soil under SiNPs.

Treatments	SOD (unit/L)	CAT (unit/L)
S0AS0	0.908 ± 0.06b	0.085 ± 0.022b
S0AS3.2	0.811 ± 0.42c	0.072 ± 0. 003b
S100As0	1.453 ± 0.16a	0.178 ± 0.018a
S100As3.2	1.502 ± 0.08a	0.183 ± 0.029a

The mean values with the same letter along the same column are not significantly different at *p* ≤ 0.05.

Table 5 shows the redox status of maize plants grown in As-contaminated soil (S0As3.2) with or without SiNPs application. It can be seen that As stress significantly reduced the GSH and oxidized GSSG in the shoots of maize plants compared to the control (S0AS0). However, the GSH/GSS ratio was the highest, which revealed the role of the GSH/GSSG ratio in maintaining the redox state of the cell^43^. The reduction in GSH under As stress could be attributed to the oxidation of GSH by ROS and biosynthesis of PCs^44^. The SiNPs decreased the activities of the non-enzymatic antioxidants, reduced GSH (Fig. 9), GSSG and GSH: GSSH.

**Table 5:** Redox status of Maize planted in Arsenic-polluted soil under SiNPs.

Treatments	GSH (mM)	GSSG (µM)	GSH/GSSG ratio
S0AS0	1.323 ± 0.011a	7.378 ± 0.240a	179.32b
S0AS3.2	0.863 ± 0.010b	3.155 ± 0.224c	273.97a
S100As0	0.330 ± 0.150c	5.389 ± 0.34b	61.23c
S100As3.2	0.112 ± 0.016d	2.087 ± 0.220d	53.67d

The mean values with the same letter along the same column are not significantly different at *p* ≤ 0.05.

### Effects of silicon nanoparticles on the synthesis of phytochelatins of maize plants under As stress

Table 6 shows the synthesis and characterization of PCs in maize roots grown in As-contaminated soil with or without SiNPs application. Arsenic stress (S0As100) induced the production of PCs in the root tissues of maize plants compared to the control (S0AS0). These results indicate that As stress induces the synthesis of PCs by activating PCS enzymes in root cells^45^. The increase in PC concentration under As stress could be attributed to the complexation of PCs with As for detoxification and sequestration purpose. The application of SiNPs boosts the synthesis of PCs (PC_2_, PC_3_, PC_4_ PC_5,_ GSSH) in the root tissues of maize plants under As stress compared to the As-stressed plants without SiNPs (S0AS3.2) (Fig. 9). The production of structural protective agents has been enhanced by SiNPs. The concentration of PCs was high under SiNPs treatment, causing inhibition of As uptake by roots and its subsequent retention in root cells by SiNPs^46^. Phytochelatins (PCs) and antioxidant enzymes are involved in the process called catalyze-redox transformations^47^. They form complexes and reduce the mobility of Arsenic in the root regions. The SiNPs=As(III)–PCs complexes were adsorbed on the cell surfaces, thereby hindering their mobility and reducing their biological reactivity. Consequently, sequester the Arsenic-ligand complex into the vacuole. PC complexes form with Arsenic stored in the vacuole and cell organelles^48^. It was observed that DesGly-PC_2_-ye and oxidized glutathione (GSSH) were more highly synthesized than other PCs components at the root. PCs are synthesized from reduced glutathione, which is one of the reasons for the decrease in the pool of intracellular glutathione^47^. This was also supported our findings in Table 5 where we observed decrease in concentration of GSH during As stress. The synthesis of PC_5_ was also attributed to the presence of Arsenic stress and SiNPs.

**Table 6:** Synthesis of phytochelatin at maize root of arsenic polluted soil under SiNPs.

Treatment	PC_2_-ye (ppm)	Desgly-PC_2_-ye	Oxidized glutathione	PC_3_-ye	Iso-PCs_3_(glu)	PC_4_-ye	PC_5_
S0AS0	0.196c	3.763c	0.119d	0.067b	0.055c	0.035c	NP
S0AS3.2	0.276b	4.665b	0.334c	0.067b	0.059c	0.038c	0.039c
S100As0	0.321a	4.990b	0.526b	0.107a	0.070b	0.064b	0.065b
S100As3.2	0.334a	5.185a	0.793a	0.144a	0.084a	0.079a	0.083a

The mean values with the same letter along the same column are not significantly different at *p* ≤ 0.05.

### Effects of silicon nanoparticles on lipid peroxidation and IC_50_ of maize plants under As stress

**Figure 7:**
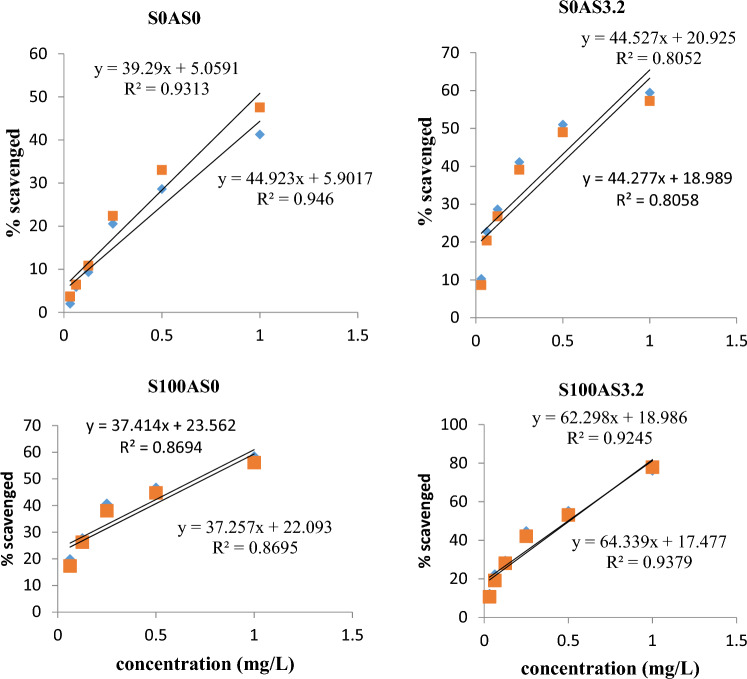
Hydroxyl Scavenged in Maize grown in Arsenic-polluted soil subjected to SiNPs, N = 36.

Figure 7 display the hydroxyl radical scavenging activity of maize roots grown in As-contaminated soil with or without SiNPs application. These results indicate that As stress (S0AS3.2) decreased the ability of shoot to scavenge hydroxyl radicals, which are one of the most reactive and damaging ROS^44^, when it was under As stress (S0AS3.2). The reduction in hydroxyl radical scavenging activity under As stress could be attributed to the depletion of antioxidants or enzymes involved in hydroxyl radical scavenging by ROS or by direct interaction with As^49^. As a result, silicon nanoparticles under As stress (S100As3.2) removed more than 70% of the OH^–^ (Fig. 7) compared to the control group (S0As3.2); this greatly decreased the amount of MDA (Table 7). We observed the balance of homeostasis and active selective ion translocation (xylem loading) of silicon instead of Arsenic from the root to the shoot in plants treated with SiNPs under As stress. SiNPs stabilized the plasma membrane and prevented As entry. They also regulated metal transporters in the root membrane, enabling simultaneous heavy metal removal^43^.

**Table 7:** Lipid peroxidation and IC50 of Maize grown on Arsenic-polluted soil under SiNPs.

	S0As0	S0As3.2	S100As0	S100As3.2
IC50	1.063 ± 0.057a	0.677 ± 0.017c	0.728 ± 0.015b	0.501 ± 0.0027d
MDA (mM)	6.91E-06 ± 8.58E-08a	4.22E-06 ± 4.49E-08b	6.8E-06 ± 4.58E-07a	3.7E-06 ± 5.72E-08c

The mean values with the same letter along the same column are not significantly different at *p* ≤ 0.05.

### Arsenic changes in plant and soil

**Figure 8:**
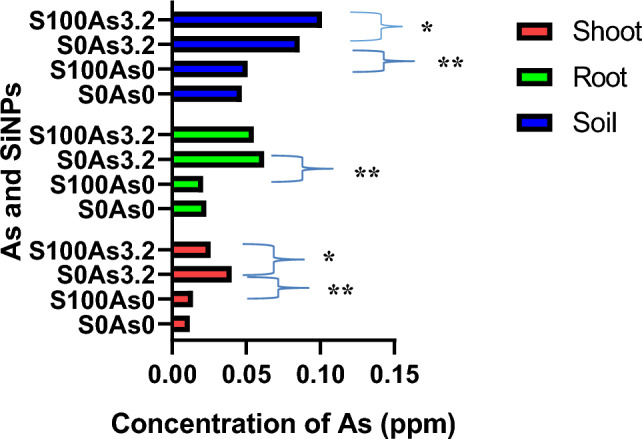
Concentration of Arsenic in Soil and Maize Tissue. * and ** represent *p*-values at 0.01 and 0.001.

**Figure 9:**
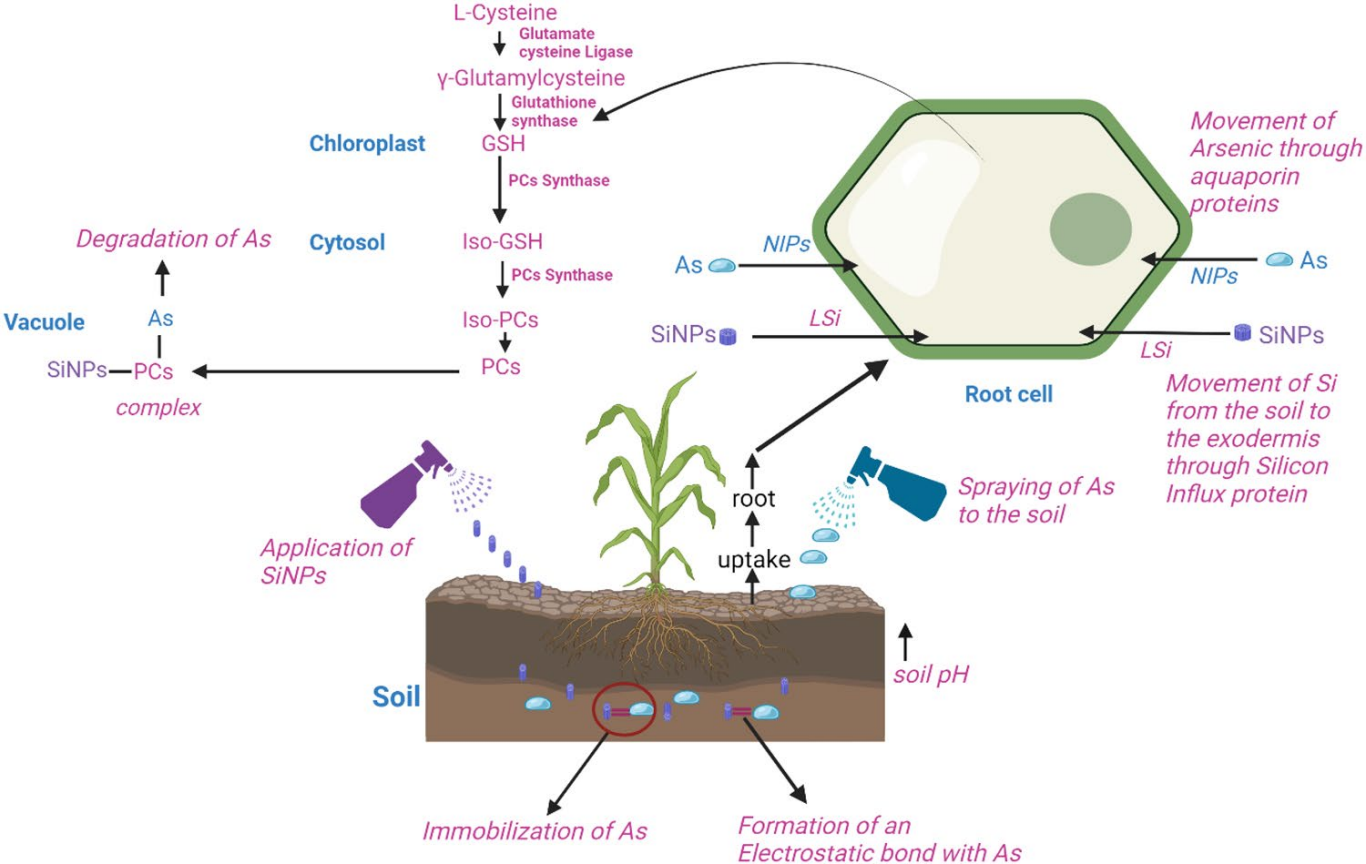
Mechanism of Arsenic Complexation and immobilization using SiNPs.

Figure 8 shows the concentration of Arsenic in the soil, root and shoot of Maize under the treatment of SiNPs. There was a retention of Arsenic in the soil and sequestration of Arsenic to the shoot of plants treated with SiNPs. The translocation of Arsenic describes the phytostabilization mechanism of reclaiming Arsenic in soil, thus allowing the maize plants to live healthy. SiNPs immobilized (As(III)=SiNPs) As in the soil through the formation of electrostatic force. Consequently, As were reduced in their bioavailability in the food chain^50^. Phosphate (Pi) transporters and nodulin 26-like intrinsic proteins (NIPs) are the major known transporters of As(V) and As(III) respectively^51^,^52^. SiNPs (S100As3.2) lessen the concentration of As in the shoot than the root, resulting in the complexation SiNPs=As(III)–PCs of As, which is ultimately sequestrated within the plant vacuoles (Fig. 9).

SiNPs in the soil formed an electrostatic bond with Arsenic which immobilized the availability of As. The presence of silicon influx protein (LSi) in Maize thus facilitates movement of SiNPs from the soil into the root exodermis. Therefore, led to the production of PCs in the cytosol as a result of decreased GSH consequently, led to the complexation and sequestration of As in the vacuole.

## Conclusion

The present study confirmed the successful synthesis of spherical, smooth, and nano-sized SiNPs from *Pycreus polystachyos*. Application of 100% SiNPs significantly enhanced the growth and yield of maize under As stress. In addition, SiNPs act as a co-enzymatic which induced the activities of antioxidant defensive system (SOD and CAT) in maize shoots under As stress. Consequently, excessive hydroxyl radicals were scavenged and cell membrane integrity were stabilized as evident in reduced lipid peroxidation. SiNPs also played a role in the accumulation of phytochelatins, especially desGly-PC2 and oxidized glutathione in maize roots under As stress. This may lead to the formation of SiNPs=As(III)–PCs complexes which reduced As mobility and sequestrated As in plant roots and shoots.

Therefore, the highlights of the beneficial effects of green synthesized SiNPs in As toxicity in maize plant includes improved growth and yield, As immobilization, complexation and sequestration, antioxidant protection and membrane stabilization. SiNPs could serve as a sustainable nano-fertilizer for enhancing productivity of maize crops grown in As contaminated soils. Further studies are warranted to understand the long-term ecological impacts of SiNPs in agroecosystems.

## Data Availability

The datasets used and/or analyzed during the current study are available from the corresponding author on reasonable request.
